# Screening and Colonoscopy Quality Measures: Ethnic Disparities and Impact on Patients' Outcome

**DOI:** 10.1155/2023/8881715

**Published:** 2023-10-30

**Authors:** Fadi Abu Baker, Dorin Nicola, Amir Mari, Abdel-Rauf Zeina, Amani Beshara, Randa Taher Natour, Yael Kopelman

**Affiliations:** ^1^Department of Gastroenterology and Hepatology, Hillel Yaffe Medical Center, Affiliated to the Technion Faculty of Medicine, Haifa, Israel; ^2^Department of Internal Medicine, Hillel Yaffe Medical Center, Affiliated to the Technion Faculty of Medicine, Haifa, Israel; ^3^Department of Gastroenterology, Nazareth EMMS Hospital, Affiliated with the Faculty of Medicine, Bar Illan University, Safed, Israel; ^4^Department of Radiology, Hillel Yaffe Medical Center, Affiliated to the Technion Faculty of Medicine, Haifa, Israel

## Abstract

**Background:**

Recent reports have confirmed the improving trends in colorectal cancer (CRC) incidence and outcomes. Still, disparities in incidence and mortality in CRC continue to persist between major ethnic groups despite the provision of widespread screening and improved care. We aimed to outline, from an endoscopic point of view, ethnic disparities in major endoscopic measures concerned with CRC screening and detection and track their impact on patients' outcomes.

**Methods:**

We reviewed electronic reports of patients referred for colonoscopy procedures over 20 years. We compared demographic, clinical, and endoscopic findings between major ethnic population groups in Israel. In addition, trends of screening utilization, bowel preparation, and polyp detection rates were tracked, and the incidence of CRC diagnosis was followed.

**Results:**

A total of 51307 patients had undergone colonoscopies, of whom 16% were Arabs, and 84% were Jewish. The procedures performed for CRC screening throughout the study period were significantly lower in Arabs (5% vs. 13.1%; *P* < 0.0001). In parallel, for most of the follow-up period, the Arab patients had higher rates of inadequate bowel preparation (overall: 19.9% vs. 12%; *P* < 0.001) and a lower polyp detection rate (16.7% vs. 22.5%; *P* < 0.0001). Expectedly, the incidence of CRC has steadily decreased in the Jewish group, while an adverse pattern of increasing incidence was documented in the Arab patient during the follow-up period.

**Conclusion:**

Characterized by lower screening utilization and poor bowel preparation, the incidence of CRC development in Arab patients is increasing, while improving trends of CRC were observed in their Jewish counterparts.

## 1. Introduction

Colorectal cancer (CRC) is a significant cause of morbidity and mortality in Israel and worldwide [1]. CRC is the third most commonly diagnosed cancer in males and the second in females and is the principal cause of gastrointestinal-related mortality [2, 3]. However, ethnic disparities in the prevalence and outcome of CRC have been observed globally, and its frequency varies remarkably among different populations [4, 5]. Indeed, the risk of developing CRC is also influenced by environmental, lifestyle, and genetic factors [6].

Fortunately, significant progress has been made in the surgical and systemic treatment of this malignant condition over the last few years. Nevertheless, only a minority of cases are diagnosed when the cancer is still localized [7]. In this regard, CRC is considered one of the cancers having the most significant benefit from preventive measures and screening programs. The classic slow progression from colonic precancerous lesions to cancer facilitates their timely detection and removal and poses a major potential for reducing the CRC burden by implementing screening programs [8]. Indeed, CRC incidence and mortality rates have declined over the past decade due to the adoption and endorsement of effective screening programs [9]. A colonoscopy is the most accurate and valuable diagnostic and therapeutic tool for detecting and removing precancerous lesions. Many societies consider it the gold standard procedure for CRC screening [10]. Several quality indicators have been established to maximize the effectiveness of colonoscopy in detecting premalignant lesions and providing high-quality endoscopic practice [11]. Among others, adequate bowel preparation, cecal intubation rate, and polyp/adenoma detection rate are considered leading quality measures that have been demonstrated to be directly associated with interval cancer [12, 13].

Like several other countries, Israel has implemented a national program for the early detection of CRC in conjunction with all local healthcare funds. This involves the annual performance of fecal occult blood tests for average-risk patients. Moreover, colonoscopy is widely used for screening, especially for high-risk patients. Consequently, recent reports have confirmed the improving trends in CRC incidence and outcomes in Israel, similar to other Western countries [14, 15]. Still, CRC disparities in incidence and outcome persist between major Israeli ethnic groups. Essentially, a distinct decrease in CRC incidence was seen in both sexes in the Jewish population, while an increase in CRC incidence in the Arab patients has occurred during the last years [16]. The reasons for these different incidence trends are unknown. They have not been widely studied but may be attributed to various sociodemographic statuses, diverse lifestyle types and healthcare utilization behaviors, and adherence to national screening programs.

In the current study, we aimed to investigate ethnic disparities in major endoscopic measures concerned with CRC screening and detection from an endoscopic point of view and to track their impact on patients' outcomes.

## 2. Methods

We conducted a retrospective, large cohort study by reviewing the electronic files of consecutive patients who had undergone colonoscopy procedures over 20 years (2000–2020) within the gastroenterology department at the Hillel Yaffe Medical Center. All patients' data were collected from our department's electronic record system. Full demographic data of patients were recorded upon admission into the endoscopy unit based on identity details and a national database. Age, sex, ethnicity, and country of birth were documented for all patients. Ethnicity was categorized based on the Israeli Central Bureau of Statistics (CBS) classification into religious ethnicity of two main groups, Arabs and Jews. Relevant clinical data, including procedures' indications, family history, and other relevant background histories, were documented. Endoscopic diagnosis and data, including bowel preparation (classified as adequate or inadequate), cecal intubation, and polyp detection rate as the main quality measures of the colonoscopy procedure, were recorded. We compared demographic, clinical, and endoscopic findings between Arab and Jewish populations.

Namely, trends of screening utilization, bowel preparation, and polyp detection rates were tracked over the study period, and the incidence of colorectal cancer development was documented. The local institutional Helsinki ethics board approved the study and granted exemption from informed consent as data collection did not influence medical practice. Helsinki ID 0195-20-HYMC.

## 3. Patient and Public Involvement

No patient is involved.

### 3.1. Statistical Analysis

Descriptive statistics was performed for comparison between the different ethnic groups of patients. Continuous parameters were presented by means ± deviations, and categorical parameters were expressed by using frequencies and percentages. Differences between both populations were compared by *t*-test in quantitative parameters and Fisher's exact test in the categorical parameters. Multivariate analysis was performed to assess the impact of ethnicity and other variables on the polyp detection rate. Curves of annual change in the bowel preparation quality and polyp detection rates were compared for both groups, and their reflection on parallel CRC incidence curves was outlined.

## 4. Results

**Figure 1:**
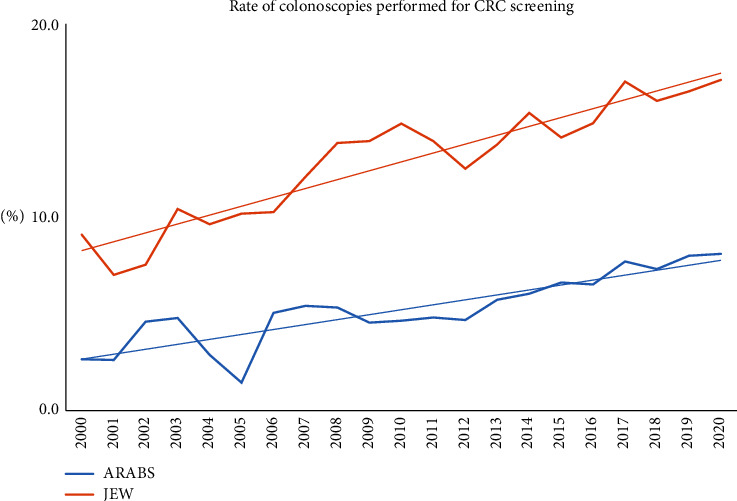
The annual rate of screening colonoscopies performed for both ethnic groups.

A total of 52113 patients had undergone colonoscopy procedures during the study period. Of these, 51307 patients met the inclusion criteria, had a complete data set, and were included for final analyses. A total of 43116 (84%) patients were Jewish, while 8191 (16%) were Arabs. No gender predominance was noted for both groups, but Arab minority patients were significantly younger at presentation (55.49 ± 15.03 vs. 60.10 ± 14.42; *P* < 0.0001). The rate of procedures performed for CRC screening throughout the study period was significantly lower in Arabs (5% vs. 13.1%; *P* < 0.0001) (Figure 1). Overall, similar rates of major colonoscopy indications were documented for both groups during the study period (Table 1).

Major endoscopic findings were significant for higher overall rates of inadequate bowel preparation (19.9% vs. 12%; *P* < 0.0001) and reduced cecal intubation rates in the Arab group (88% vs. 83.8%; *P* < 0.001), while terminal ileum intubation did not differ between the groups. Moreover, the polyp detection rate (16.7% vs. 22.5%; *P* < 0.0001) was significantly lower for the Arab patients compared to their Jewish counterparts. In the multivariate analysis, advanced age, male sex, and bowel preparation were independently and significantly associated with PDR, while ethnicity was not linked to PDR (OR 1.2, 95% CI 0.85–1.42; *P* = 0.22).

**Figure 2:**
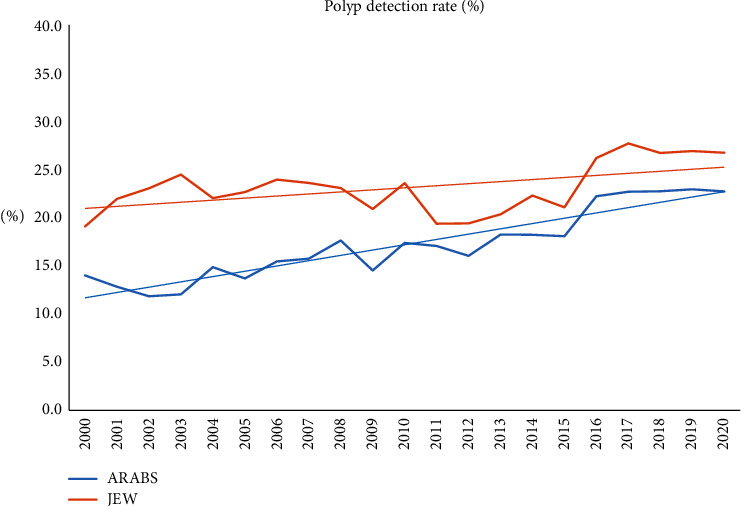
The annual polyp detection rate for both ethnic groups.

**Figure 3:**
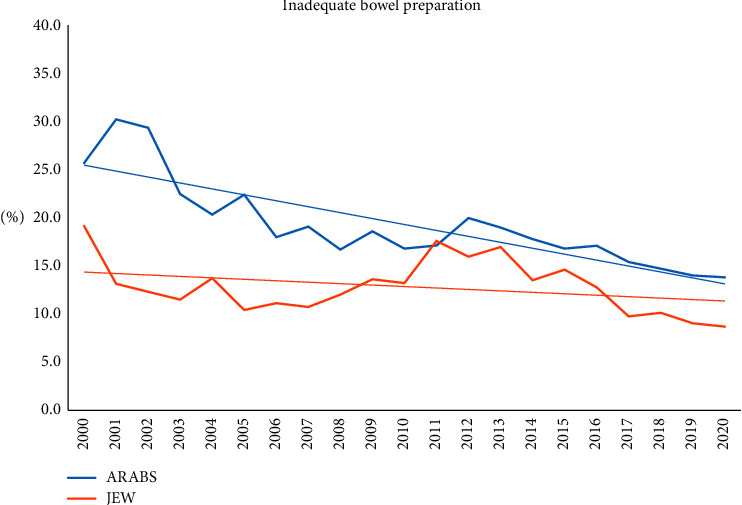
The annual rate of poor bowel preparation for both ethnic groups.

The follow-up of major endoscopic findings throughout the study period revealed that the Arab patients had higher rates of inadequate bowel preparation and lower polyp detection rates for most of the follow-up period. However, improving trends of bowel preparation and increasing PDR were steadily documented over time for both groups, particularly for the Arab patients (Figures 2 and 3). Moreover, the referral for screening procedures has progressively increased over time for both groups but was still significantly higher for Jewish patients.

**Figure 4:**
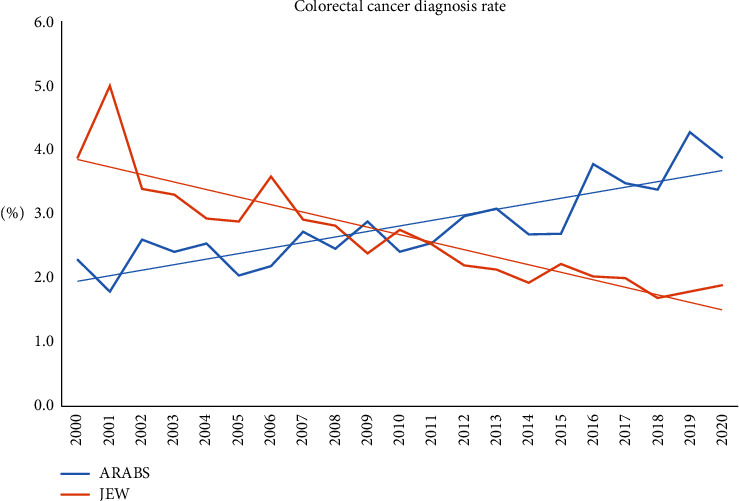
The annual cancer diagnosis rate for both ethnic groups.

Interestingly, the incidence of CRC has steadily decreased in the Jewish group. In contrast, a negative pattern of increasing incidence was documented in the Arab patients during the follow-up period (Figure 4).

## 5. Discussion

The incidence and mortality rates of CRC, one of the most common tumors in Israel and worldwide, have declined over the past decade due to the adoption and increased endorsement of effective screening programs [9, 17].

Still, ethnic disparities in the presentation, incidence, and outcome of CRC have been reported in several studies [18–20]. Besides biological differences and environmental factors, healthcare utilization behaviors, medical-seeking manners, and adherence to national screening programs may have a crucial impact [21]. Performing high-quality endoscopy is mandatory for the detection and removal of precancerous lesions. Still, major quality measures for colonoscopy performance may differ between ethnic groups contributing to a varied CRC incidence and outcome [22]. By utilizing data from two large cohorts of different ethnic populations, we aimed to investigate the effect of screening trends and major endoscopic measures on CRC incidence and outcome from an endoscopic point of view.

First, we demonstrated that significantly higher rates of Jewish patients were referred for screening colonoscopy for most of the follow-up period than Arab minority patients. Even though improving trends of screening utilization were documented for both populations, a significant gap still exists, and the referral for screening in the Arab population is far from ideal. This may indicate a low public awareness and education and should be managed by addressing the general population as well as primary care providers. The use of media and social networking to provide culturally appropriate education to the potential population in their native language is one of the foremost needed measures.

Second, from the endoscopic point of view, we demonstrated that a poor fulfillment of major colonoscopy quality indicators characterized the Arab minority patients. Namely, higher rates of inadequate bowel preparation combined with reduced cecal intubation rate and low PDR were documented. In this regard, it is well established that an inadequate level of bowel preparation may increase the risk of adverse events related to the procedure, lengthen the procedure time, and be inversely related to cecal intubation and adenoma detection rates [23, 24]. This was obvious in the multivariate analysis demonstrating that inadequate bowel preparation was significantly and independently associated with reduced PDR but not linked with ethnicity.

Fortunately, we did show an improved trend of major quality indicators over the last years for both ethnicities, but this positive change is still insufficient and below the accepted standards. Therefore, education and measures to improve preparation are extremely important, and further study to investigate risk factors of poor preparation in this population is warranted.

In parallel, we demonstrated that the trends of CRC detection are decreasing in the Jewish population, while it is on the rise in their Arab counterparts. Our findings concordance with national data indicating that the trends in the Jewish population showed an increased incidence up to the year 1994, a plateau from 1994 to 2006, followed by a slight decrease, while the Arab population showed a continuous increase in disease incidence [25]. These results are surprising as the Arabic society had undergone and maintained a significant modernization process and shared similar geographical areas and environmental factors with Jews. In addition, recent data indicate that the socioeconomic gap between the two ethnicities has been significantly reduced in the last two decades [26–28]. Despite the fact that many other factors may have contributed to these variances in CRC epidemiology, we believe that our findings demonstrating low screening utilization, suboptimal bowel preparation, and reduced PDR have a major contribution to these differences and are of great value, being potentially modifiable factors.

Thus, encouraged by the improving trends of bowel preparation, polyp detection, and screening utilization shown in the current study during the last years for both groups, we believe it is time for collaboration and concerted efforts to increase awareness and facilitate the design and implementation of appropriate public health policies and interventions to minimize these racial disparities.

Above all, we believe that our findings, as translated into the different outcomes of two ethnicities, can be generalized to highlight the paramount significance and the clinical value of the colonoscopy's quality indicators as predictors of high-quality endoscopic practice, directly associated with CRC detection and prevalence figures. These quality measures should be rigorously documented and followed to meet the accepted standards, particularly in minority populations.

Limitations of the current study are inherent in its retrospective and single-center design. Moreover, as the present study was aimed at investigating the endoscopic perspective, relevant data on confirmed CRC risk factors such as family history, smoking, and obesity, among others, were lacking and may have affected CRC outcome and incidence.

## 6. Conclusion

We outlined major ethnic disparities concerned with screening utilization and colonoscopy quality indicators and demonstrated their direct and long-term impact on CRC incidence.

## Figures and Tables

**Table 1:** Baseline characteristics of both groups.

Ethnic group	Arabs; *n* = 8191	Jews; *n* = 43116	*P* value
*Epidemiological data*			
Age (average SD)	55.49 ± 15.03	60.10 ± 14.42	*P* < 0.0001
Gender (male)	4424 (54%)	21659 (50.2%)	*P* < 0.0001

*Procedures' indications*			
Abdominal pain diarrhea	2423 (29.6%)	10661 (24.7%)	*P* < 0.0001
Iron deficiency anemia	1475 (18.0%)	7675 (17.8%)	*P* = 0.07
Positive FOBT	632 (7.7%)	3277 (7.6%)	*P* = 0.12
Rectal bleeding	1157 (14.1%)	6333 (14.7%)	*P* = 0.19
Screening	404 (5%)	5638 (13.1%)	*P* < 0.0001
Constipation	956 (11.7%)	4557 (10.6%)	*P* = 0.003
Suspicious imaging finding	395 (4.8%)	1966 (4.6%)	*P* = 0.066
Weight loss	274 (3.3%)	1259 (2.9%)	*P* = 0.039

*Endoscopic findings*			
Cecal intubation rate	6681 (83.3%)	36987 (89.0%)	*P* < 0.0001
Terminal ileum intubation	288 (3.5%)	1396 (3.2%)	*P* = 0.07
Bowel preparation (poor)	1627 (19.9%)	5179 (12%)	*P* < 0.0001
Polyp detection rate (overall)	1365 (16.7%)	9688 (22.5%)	*P* < 0.0001
CRC detection rate (overall)	235 (2.9%)	1159 (2.6%)	*P* = 0.09

## Data Availability

The data supporting this study's findings are available on request from the corresponding author, RTN.
